# Analysis of temporal fecal microbiota dynamics in weaner pigs with and without exposure to enterotoxigenic *Escherichia coli*^1^,^2^

**DOI:** 10.1093/jas/sky260

**Published:** 2018-07-03

**Authors:** Jolinda Pollock, David L Gally, Laura Glendinning, Raksha Tiwari, Michael R Hutchings, Jos G M Houdijk

**Affiliations:** 1 Animal and Veterinary Sciences, Scotland’s Rural College (SRUC), Edinburgh, UK; 2 The Roslin Institute and Royal (Dick) School of Veterinary Studies, University of Edinburgh, Edinburgh, UK; 3 Research and Development, Zoetis, Kalamazoo, MI, USA

**Keywords:** 16S rRNA gene, ETEC, metabarcoding, microbiome, weaner pigs

## Abstract

The primary aim of this work was to study potential effects of subclinical enterotoxigenic *Escherichia coli* (**ETEC**) exposure on porcine fecal microbiota composition, with a secondary aim of profiling temporal shifts in bacterial communities over the weaning transition period. 16S rRNA gene metabarcoding and quantitative PCR (**qPCR**) were used to profile the fecal microbiota and quantify ETEC excretion in the feces, respectively. Temporal shifts in fecal microbiota structure and stability were observed across the immediate postweaning period (*P* < 0.05), including significant shifts in the relative levels of specific bacterial phylotypes (*P* < 0.05). ETEC exposure did not change the fecal microbiota structure (*P* > 0.05), but significant variations in fecal community structure and stability were linked to variations in ETEC excretion level at particular time points (*P* < 0.05). In this study, marked temporal changes in microbiota structure and stability were evident over the short weaning transition period, with a relationship between ETEC excretion level and fecal microbiota composition being observed. This study has provided a detailed analysis of fecal microbiota dynamics in the pig, which should help to inform the development of novel management strategies for enteric disorders based on an improved understanding of microbial populations during the challenging postweaning period.

## INTRODUCTION

The importance of the gut microbiota in health and development is well documented in the pig (Leser et al., 2002; Isaacson and Kim, 2012), with marked changes in bacterial composition being observed throughout the production cycle (Kim et al., 2012; Holman and Chénier, 2014; Mach et al., 2015). The emergence of next-generation sequencing methodologies, such as 16S rRNA gene metabarcoding, now provides the opportunity to study complex microbial communities with high resolution. After weaning in pigs, there is an increased risk for the development of enteric disorders such as postweaning colibacillosis. The symptoms that present as part of postweaning colibacillosis range from fecal shedding of enterotoxigenic *Escherichia coli* (**ETEC**) (Fairbrother et al., 2005; Luppi et al., 2016) with no diarrhea to peracute fatal diarrhea (Hodgson and Barton, 2009). The disease can be present at a subclinical level, whereby diarrhea is absent, but a variable reduction in performance may occur postweaning (Hampson, 1994), which depending on its magnitude can clearly be of economic importance. In this study, a previously developed subclinical ETEC exposure model (Athanasiadou et al., 2010) and 16S rRNA gene sequencing and quantitative polymerase chain reaction (**qPCR**) were utilized to assess the impact of ETEC exposure on fecal microbiome composition and ETEC shedding dynamics. In addition, temporal changes in microbiome composition were assessed over the weaning transition period. There are published studies that describe changes in the fecal microbiota during the weaning transition period specifically (Hu et al., 2016; Chen et al., 2017), but to our knowledge, this is the first study that focusses on the impact of ETEC exposure on the fecal microbiota using 16S rRNA gene metabarcoding.

## MATERIALS AND METHODS

The animal experiment described was reviewed and approved by SRUC’s Animal Welfare and Ethical Review Body (ED AE 23–2013) and carried out under Home Office regulations (PPL 60/4489).

### Pigs and Housing

Fifty-nine pigs (Large White × Landrace) were weaned at 26.7 ± 0.7 d of age (mean ± SD) and weighed 8.65 ± 1.77 kg, with 27 pigs being used in Round 1 (June 2013) and 32 pigs being used in Round 2 (August 2013). Pens were balanced as much as possible for sex, weaning weight, and litter origin, with 8 litters being included across the trial. Pigs were housed in 4 m^2^ pens in groups of 4 maximum. The pens were bedded with sawdust as required, and a single feeder and nipple drinker were included. Water and feed were provided ad libitum for the trial duration. The environmental temperature was set at 25 °C for the first 4 d and was decreased by 2 °C per week for the experiment duration. The shed lights were switched on between 0800 and 1800 h, and night lights were maintained between 1800 and 0800 h. Pigs were fed a standard industry weaner diet for the first 14-d postweaning (digestible energy 16.9 MJ/kg; lysine 16.7 g/kg), before being moved onto a second phase weaner diet for the remainder of the trial (digestible energy 15.1 MJ/kg; lysine 15.0 g/kg), both of which did not contain antibiotics, organic acids, or supranutritional levels of zinc oxide.

### ETEC Inoculum Preparation

An ETEC O149:K91:F4 (ETEC F4) strain isolated from a weaner pig diagnosed with clinical postweaning colibacillosis (SAC Veterinary Services, UK) was incubated in brain-heart infusion broth for 24 h at 37 °C in an orbital shaker. Bacterial cells were harvested by centrifugation, and the pellet was washed 3 times in 25 mL of sterile PBS. The pellet was then resuspended in 30 mL of PBS before preparation of an inoculum containing an estimated 10^8^ cfu/mL. The optical density of the inoculum was measured using a spectrophotometer to estimate the concentration of ETEC cells. The inoculum was also serially diluted and enumerated on MacConkey agar for more accurate post hoc confirmation of bacterial concentration.

### ETEC Exposure

Thirty-two pigs (16 in each of the 2 rounds) were administered 10^8^ cfu of ETEC in PBS at 5 time points (days 4, 6, 8, 11, and 13 postweaning) as previously described (Athanasiadou et al., 2010), with weaning day defined as day 0. Briefly, 10 mL of the final inoculum was mixed with a further 10 mL of sterile PBS, before mixing with 20 g of feed. This mixture was then offered in small, discrete bins for each pig, with individual dosing being facilitated by temporarily splitting the pens in 2 for paired feeding. The remaining 27 sham-exposed (control) animals (11 in Round 1 and 16 in Round 2) were provided with feed in the same manner, mixed with 20 mL of sterile PBS only.

### Fecal Sampling and DNA Extraction

Fecal samples were taken directly from the rectum on day 4 (before ETEC exposure), days 8 and 12 (during ETEC exposure), and days 15 and 19 (after ETEC exposure) using a spooned universal tube. The samples were immediately snap-frozen on dry ice prior to storage for a maximum of 2 wk at −80 °C. DNA extraction was carried out using the MoBio PowerSoil DNA Isolation kit (Cambio, UK), with modifications to the protocol. Briefly, 500 mg of feces was transferred into a 50-mL centrifuge tube, and 5 mL of MoBio PowerSoil Bead Solution (Cambio, UK) was added to each tube. The feces was then homogenized using a vortex, and 1 mL of supernatant was transferred into the provided bead tube, before being homogenized for 45 s at 5.0 m/s using a FastPrep FP120 Cell Disrupter (Qbiogene Inc, France). The homogenate was then processed according to the included kit protocol. The yield and quality of the DNA extracts were tested using a NanoDrop spectrophotometer (Thermo Scientific, UK) and by running the extracts on a 2% agarose gel. The agarose gel visualization confirmed the presence of intact, high-molecular-weight DNA.

### ETEC Quantification

Fecal excretion of ETEC F4 was determined using qPCR, which targeted the *faeG* major fimbrial subunit. Reactions were set up using Brilliant III Ultra-Fast SYBR Green qPCR Master Mix (Agilent Technologies) and primers F4-463F (5′-GGTTCTGAACTCTCGGCTGG–3′) and F4-597R (5′-AGAACCTGCGACGTCAACAA-3′), which were designed as part of this study. All reactions were carried out in triplicate using a Stratagene MX3005P instrument (Agilent Technologies), with 2 µL of DNA extract being added to each reaction. The qPCR run conditions consisted of an initial denaturation step at 95 °C (5 min), followed by 40 cycles of amplification at 95 °C (30 s) and then at 65 °C (15 s). A melt curve was generated using the following cycling conditions: 95 °C (60 s), 55 °C (30 s), and 72 °C (30 s). A subsample of purified PCR products from ETEC-exposed animals were sequenced to test the specificity of the designed primers (Eurofins, Germany), and the sequences were then matched using the NCBI BLAST reference database (http://blast.ncbi.nlm.nih.gov/Blast.cgi).

To enable calculation of target gene copy number in the fecal samples, absolute quantification using a standard curve was carried out. The standard curve was constructed using purified PCR products from tenfold serial dilutions of the ETEC F4 strain. To convert the quantity given by the qPCR output to the number of *faeG* gene copies, it was calculated that one nanogram of DNA contained 6.86 × 10^9^ copies of the target gene. The original concentration of the standards was determined using a spectrophotometer (NanoDrop 1000, Thermo Scientific, UK), and these values were used to estimate the number of gene copies per gram of wet feces.

### Pig Selection for DNA Amplification and Sequencing

Thirty-two pigs were selected for 16S rRNA gene metabarcoding analysis (16 ETEC-exposed and 16 control pigs) (Supplementary Appendix 1), including 1 sample pre-exposure and 4 samples postexposure. Weaning weight, sex, litter origin, and experimental round were considered when recruiting particular pigs to the 16S rRNA gene metabarcoding study.

The V3 hypervariable region of the 16S rRNA gene was amplified using dual- indexed primers, which were previously used during a pig gut microbiome study—341F (5′- CCTACGGGAGGCAGCAG-3′) and 518R (5′- ATTACCGCGGCTGCTGG-3′) (Kim et al., 2012), which incorporated TruSeq adapters (Supplementary Appendix 2). Template DNA was amplified in a 1-step PCR using a high fidelity polymerase (Phusion, New England Biolabs, UK). A PCR mastermix was constructed to carry out 20-µL reactions, including primers at a final concentration of 0.2 µM. The PCR conditions consisted of an initial denaturation step at 98 °C (3 min), followed by 20 cycles of amplification at 98 °C (30 s), 60 °C (30 s), then 72 °C (30 s), and a final extension step at 72 °C (5 min). PCR products were purified using the AMPure XP PCR purification system (Beckman Coulter).

Reagent-only controls were amplified in parallel by adding 5 µL of DNA extract to the PCR reaction mixture. The Human Microbiome Project mock community HM-782D (BEI Resources, ATCC, Manassas, VA) was also amplified by adding 1 µL of preprepared DNA extract (containing 100,000 16S rRNA gene copies per organism per µL) to the PCR reaction mixture. The presence of the correct sized product was confirmed by gel electrophoresis and by use of a TapeStation instrument (Agilent Technologies, UK). Before submission for sequencing (Edinburgh Genomics, UK), double-stranded DNA was quantified using a fluorometric assay (Qubit dsDNA HS Assay kit, Invitrogen, UK). Readings from this assay were used to create 2 pools (80 samples per pool), using equimolar concentrations of each library. Sequencing was carried out using the Illumina MiSeq platform (Illumina, CA), using V2 chemistry and producing 250-bp paired-end reads.

### Sequence Processing

Primer sequences were removed from raw sequence files using cutadapt (Martin, 2011). The following processing steps were carried out using the open source software, mothur (Schloss et al., 2009), based on a protocol written by the developers (Kozich et al., 2013) (URL: https://www.mothur.org/wiki/MiSeq_SOP. Accessed December 2014). Briefly, contiguous sequences were constructed from the paired-end reads. These sequences were then aligned to reference sequences from the SILVA small-subunit rRNA sequence database (Pruesse et al., 2007), and those that did not map to the correct position in this file were removed. Sequences were also removed if they were below 135 bp in length or above 230 bp in length, if they contained over 8 homopolymers, and if they contained ambiguous bases. Chimeras were identified and removed using UCHIME (Edgar et al., 2011). Sequences were classified using the Greengenes database (DeSantis et al., 2006), which was trimmed to the V3 hypervariable region of the 16S rRNA gene to improve classification depth (Werner et al., 2012). Sequences that did not originate from bacteria were removed. The remaining sequences were binned into phylotypes based on their similarity to reference sequences and were subsampled for analysis.

### Pig Growth Rate and Fecal Consistency Scores

All pigs were weighed on days 0, 7, 14, 21, and 28 to assess growth rate over the trial duration. Consequently, the ADG per pig was calculated over the trial duration. The general health and cleanliness of each pig was closely monitored and scored for the duration of the experiment. Throughout the experiment, pigs remained in good health, measured by active response to human presence and by the presence of pink skin, bright eyes, and upright ears. Fecal consistency scores were recorded daily as described previously (Wellock et al., 2006) on a pen basis using a subjective four-point scale (1, normal; 2, normal diarrhea; 3, watery diarrhea; and 4, dysentery).

### Descriptive and Statistical Analysis of Sequence Data

Descriptive and statistical analyses were carried out to describe temporal microbiota shifts and to establish whether there was an effect of ETEC exposure on the fecal microbiota and/or a link between ETEC excretion level and fecal microbial communities. Analyses were carried out using the mothur software package (Schloss et al., 2009) unless stated otherwise.

The Inverse Simpson’s Index (**ISI**) was calculated for each sample to measure diversity, and the Chao 1 index was calculated to assess richness. To test whether there were significant differences in diversity and richness over time and between ETEC-exposed and control pigs, repeated measures ANOVA (**RM-ANOVA**) was carried out using Genstat 16 (VSN International, UK). The values for day 4 were initially included as covariates, but these had no significant effect and were therefore not included as covariates in the final analysis. Temporal changes in relative abundances at both phylum and family levels were also assessed using RM-ANOVA with logit-transformed data.

A distance matrix was compiled using Yue and Clayton theta similarity coefficients (Yue and Clayton, 2005), which take into account both community membership and relative abundance. Non-metric multidimensional scaling (**NMDS**) plots were constructed in 2D with coordinates generated using the NMDS function to visualize community similarities over time and between groups. The statistical significance of any clustering was assessed by analysis of molecular variance (**AMOVA**) (Excoffier et al., 1992). The statistical significance of variation between populations was assessed using homogeneity of molecular variance (**HOMOVA**) (Stewart and Excoffier, 1996).

To identify phylotypes that were expressed significantly different between sample groups, Metastats (Paulson et al., 2011) and analysis of composition of microbiomes (**ANCOM**; Mandal et al., 2015) tools were used, and the *P*-values were corrected for multiple observations.

To assess whether there were temporal effects of ETEC excretion level on microbiota composition, pigs were clustered into groups based on ETEC shedding level as measured by qPCR. To assess potential links between ETEC excretion level and microbiota structure, stability and phylotype relative abundances, AMOVA, HOMOVA, Metastats and ANCOM tools were used.

### Statistical Analysis of Growth Rate and Fecal Consistency Score Data

Statistical analyses were carried out using Genstat 16 (VSN International, UK) unless stated otherwise. The BW data were assessed using RM-ANOVA to establish any temporal effects of ETEC exposure. This analysis included ETEC exposure as a main factor and experimental round as a block. Day 0 values for BW were used as covariates for assessment of changes in BW. The ADG data were assessed using ANOVA to establish whether ETEC exposure had an effect on total weight gain between days 0 and 28. To assess the consistency over time of the fecal scores, and whether there were any effects of ETEC exposure, an ordinal logistic regression (**OLR**) was performed using Minitab 17 (Minitab Inc). The categorical indicator (i.e., fecal consistency score) was assigned as the response, and time point and ETEC exposure status were assigned as categorical predictors.

## RESULTS

### Sequencing Quality Control

After removing poor-quality sequences and sequencing artifacts (17% of the original reads), a total of 16,816,541 reads were left for analysis. On average, 109,434 ± 43,035 (mean ± SD) reads were analyzed per sample, and 590 phylotypes were identified, with 90% of reads being classified at phylum level, 68% at family level, 51% at genus level, and 24% at species level.

To ensure that sequencing depth was adequate for this study, Good’s coverage was calculated. All samples had an estimated Good’s coverage over 0.99, indicating that an estimated 99% of the bacteria present in the fecal samples were captured during sequencing.

Using the mock community data, the sequencing error rate was calculated as 0.03%. All bacteria in the mock community were identified to genus level, and 45% of the strains were identified at species level. The proportions of expected and measured relative abundances are highlighted in Table 1. *Acinetobacter baumannii*, *Bacillus cereus*, and the streptococci were under-represented by sequencing, whereas *Clostridium beijerinckii* was over-represented.

**Table 1.** Measured and expected relative abundances of mock community strains. List of bacterial strains included in the mock bacterial community (including 2 *Staphylococcus* and 3 *Streptococcus* species), the obtained level of taxonomic classification postsequencing, and both measured and expected relative abundances

Mock community strain(s)	Level of identification	Relative abundance (%)	
		Measured	Expected
*Acinetobacter baumannii*	Genus	0.01	5.00
*Actinomyces odontolyticus*	Genus	4.99	5.00
*Bacillus cereus*	Species	0.01	5.00
*Bacteroides vulgatus*	Genus	6.52	5.00
*Clostridium beijerinckii*	Genus	13.83	5.00
*Deinococcus radiodurans*	Genus	4.20	5.00
*Enterococcus fecalis*	Genus	5.19	5.00
*Escherichia coli*	Species	6.97	5.00
*Helicobacter pylori*	Species	8.89	5.00
*Lactobacillus gasseri*	Genus	6.24	5.00
*Listeria monocytogenes*	Species	8.00	5.00
*Neisseria meningitidis*	Genus	6.51	5.00
*Propionibacterium acnes*	Species	8.05	5.00
*Pseudomonas aeruginosa*	Genus	2.48	5.00
*Rhodobacter sphaeroides*	Species	2.78	5.00
*Staphylococcus* spp.	Species	13.41	10.00
*Streptococcus* spp.	Genus	5.68	15.00

Low DNA yield was evident from the reagent-only control extracts according to spectrophotometer measurements (NanoDrop 1000, Thermo Scientific, UK); however, background DNA contamination was detected postsequencing. These sequences were diverse with low read numbers within each phylotype.

### Taxonomic Classification of Sequences

A total of 21 different phyla were identified across all fecal samples. The majority of sequences were members of the Bacteroidetes (46%) or Firmicutes (34%), comprising 80% of all sequences. Spirochaetes and Proteobacteria were the third and fourth most dominant phyla, each comprising 4% of the total number of sequences. At family level, 50% of all sequences belonged to (in decreasing order of abundance) the Prevotellaceae, Lachnospiraceae, Ruminococcaceae, Paraprevotellaceae, and Veillonellaceae.

A phylotype-based analysis was carried out, whereby sequences were binned according to taxonomic information. A total of 590 bacterial phylotypes were identified, with 14 of these phylotypes each representing over 1% of the total number of sequences. The most abundant phylotype was assigned as *Prevotella*, comprising 25% of the total number of sequences.

There were no effects of experimental round, weaning day weight, or sex on microbiota structure or phylotype relative abundances at any time point (*P* > 0.05). Therefore, data from both experimental rounds were combined for analysis.

### Temporal Changes in the Fecal Microbiota

**Figure 1.**
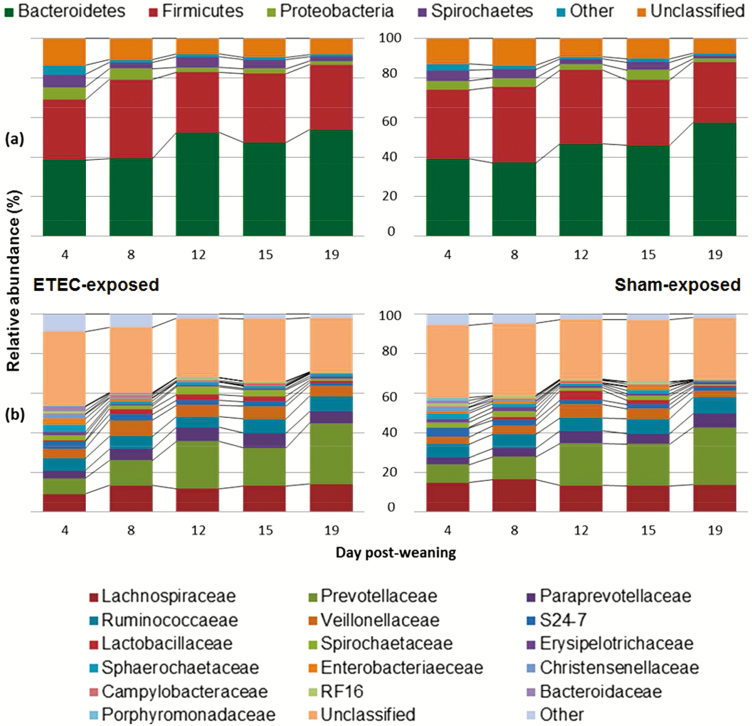
Mean phylum- and family-level shifts in relative abundance: The bacterial (a) phyla and (b) families identified in highest mean abundances, showing changes in relative abundances over a 19-d period postweaning in both ETEC-exposed and sham-exposed pigs.

The changes in relative abundances of dominant phyla and families are illustrated in Fig. 1. A significant increase in relative abundance was observed at phylum level in the Bacteroidetes (RM-ANOVA: *P* < 0.001), with significant decreases in relative abundance in both the Proteobacteria and Spirochaetes (RM-ANOVA: *P* < 0.05). There were no significant temporal changes in relative abundances at family level (RM-ANOVA: *P* > 0.05). In addition, the richness (Chao 1) and diversity (ISI) of the fecal microbiota decreased significantly from days 4 (Chao 1 = 74.31 ± 10.11, ISI = 7.09 ± 2.23) to 19 (Chao 1 = 65.29 ± 8.58, ISI = 5.57 ± 1.72) of the trial, inclusive of all pigs (RM:ANOVA: *P* < 0.01; Table 2).

**Table 2.** Mean richness (Chao 1) and diversity [Inverse Simpson’s Index (ISI)] metrics. Alpha diversity of communities associated with ETEC-exposed (ETEC) and sham-exposed (Sham) pigs with temporal changes being assessed by repeated measures ANOVA

Day postweaning	Richness (Chao 1) (± SD)		Diversity (ISI) (± SD)	
	ETEC	Sham	ETEC	Sham
4	72.88 ± 11.39	75.64 ± 8.92	6.61 ± 2.24	7.54 ± 2.20
8	65.49 ± 7.95	76.22 ± 14.33	8.84 ± 1.63	8.43 ± 2.18
12	68.67 ± 11.90	68.63 ± 10.51	7.14 ± 2.31	6.87 ± 1.74
15	70.25 ± 11.01	70.44 ± 8.96	7.84 ± 1.97	7.15 ± 2.30
19	66.42 ± 9.63	64.16 ± 7.54	5.44 ± 1.58	5.45 ± 1.90
*P*-value				
Time	0.008		<0.001	
Exposure	0.242		0.918	
Time × exposure	0.050		0.415	

**Figure 2.**
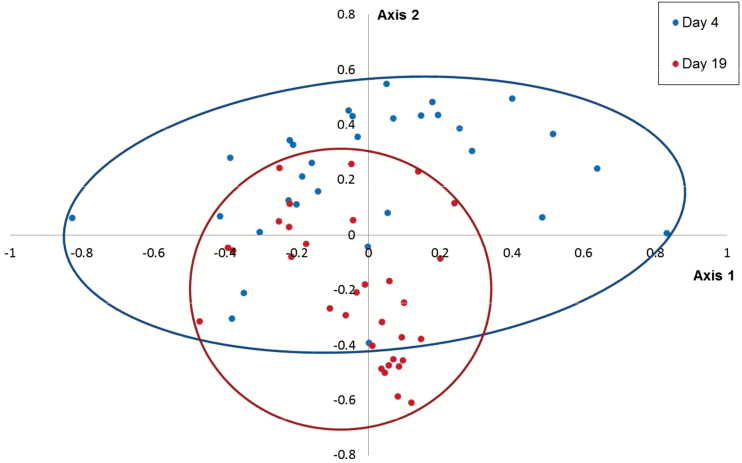
NMDS ordination comparing fecal communities on days 4 (pre-ETEC exposure) and 19: Both ETEC- and sham-exposed pigs are included to visualize shifts in overall microbiota structure by comparing baseline samples (day 4) with the final samples (day 19). Shifts in community structure (AMOVA: *P* < 0.05) and a decrease in genetic diversity (HOMOVA: *P* < 0.05) were evident over time. AMOVA = analysis of molecular variance; ETEC = enterotoxigenic *Escherichia coli*; HOMOVA = homogeneity of molecular variance; NMDS = non-metric multidimensional scaling.

There was a change in fecal microbial community structure over time when considering both ETEC-exposed and control pigs (AMOVA: *P* < 0.05). In addition, over the postweaning period, the stability of the microbiota increased in both experimental groups (HOMOVA: *P* < 0.05). This is also visualized in a simplified NMDS plot, which combines all samples from days 4 (baseline) to 19 (Fig. 2).

A small number of phylotypes were differentially expressed between days 4 and 8 in ETEC-exposed pigs (unclassified Clostridiales, *Prevotella* and Erysipelotrichaceae, Metastats: *P* < 0.05) and in control pigs (*Prevotella copri*, *Lactobacillus*, *Fecalibacterium prausnitzii*, and Erysipelotrichaceae, Metastats: *P* < 0.05). No significant changes in phylotype relative abundances occurred between days 8 and 12 and between days 12 and 15 in both ETEC-exposed and control pigs (Metastats: *P* > 0.05). Further changes in phylotype relative abundances occurred between days 12 and 19, with a decrease in *Lactobacillus* being evident in both ETEC-exposed and control pigs (Metastats: *P* < 0.05). Changes in phylotype relative abundances between days 4 and 19 have been summarized in Table 3. Phylotypes identified as *Prevotella copri*, *Prevotella stercorea*, and *Prevotella* showed significant increases in relative abundance in both experimental groups (Metastats: *P* < 0.05). *Fecalibacterium prausnitzii* and Erysipelotrichaceae also showed a significant increase in relative abundance in both experimental groups (Metastats: *P* < 0.05). The relative abundance of S24-7 (family) decreased significantly in both groups of pigs (Metastats: *P* < 0.05).

**Table 3.** Mean changes in relative abundances (± SEM) of dominant OTUs between days 4 and 19. Changes were tested using Metastats^1^ in both ETEC- and Sham-exposed pigs. The OTU relative abundance cutoff was assigned at 0.1% for any given time point

	ETEC			Sham		
Phylotype	Day 4	Day 19	*P*-value	Day 4	Day 19	*P*-value
Unclassified Bacteria	14.5 ± 2.8	8.0 ± 1.0	0.077	12.9 ± 1.9	7.6 ± 0.7	**0.001**
Unclassified Lachnospiraceae	7.8 ± 1.0	7.5 ± 1.4	0.787	13.6 ± 2.2	6.7 ± 0.6	**0.001**
*Prevotella copri*	4.7 ± 1.5	21.6 ± 2.8	**0.006**	6.2 ± 2.0	20.0 ± 3.2	**0.001**
Unclassified Clostridiales	4.1 ± 0.6	2.5 ± 0.4	0.059	3.5 ± 0.4	2.6 ± 0.4	**0.015**
*Prevotella*	1.7 ± 0.3	6.1 ± 1.7	**0.006**	1.8 ± 0.5	5.7 ± 0.9	**<0.001**
Unclassified Bacteroidetes	2.8 ± 0.7	3.6 ± 0.4	0.403	2.2 ± 0.4	3.5 ± 0.5	**0.006**
[Prevotella]	2.3 ± 1.1	3.9 ± 0.7	0.270	2.3 ± 0.7	4.9 ± 0.6	**0.002**
S24-7 (Bacteroidetes)	3.5 ± 0.8	1.4 ± 0.3	**0.010**	4.7 ± 1.3	1.8 ± 0.4	**0.002**
*Prevotella stercorea*	1.2 ± 0.4	3.3 ± 0.5	**0.009**	0.9 ± 0.2	2.9 ± 0.3	**<0.001**
Unclassified Ruminococcaceae	2.5 ± 0.5	2.1 ± 0.2	0.485	3.1 ± 0.5	2.2 ± 0.3	**0.015**
*Anaerovibrio*	2.8 ± 1.5	2.5 ± 0.5	0.818	1.8 ± 0.5	1.3 ± 0.2	**0.040**
*Lactobacillus*	0.4 ± 0.3	0.3 ± 0.1	0.838	0.0 ± 0.0	0.2 ± 0.0	**0.002**
*Treponema*	2.6 ± 1.0	1.0 ± 0.5	0.229	2.5 ± 0.6	1.0 ± 0.3	**0.006**
*Phascolarctobacterium*	1.3 ± 0.2	1.2 ± 0.2	0.647	1.4 ± 0.2	0.8 ± 0.2	**0.006**
Sphaerochaeta	3.5 ± 0.9	1.3 ± 0.5	0.067	2.9 ± 0.6	0.8 ± 0.2	**0.001**
*Fecalibacterium prausnitzii*	0.4 ± 0.3	2.2 ± 0.6	**0.009**	0.3 ± 0.1	2.9 ± 0.7	**<0.001**
Erysipelotrichaceae	0.0 ± 0.0	3.5 ± 0.7	**0.006**	0.1 ± 0.1	3.6 ± 0.6	**<0.001**

^1^Statistically significant shifts in phylotype relative abundances are highlighted in bold (false discovery rate corrected *P*-value: *P* < 0.05).

When considering all time points in the longitudinal analysis, there were no consistently differentially expressed phylotypes over the course of the experiment (ANCOM: *P* > 0.05).

### ETEC Quantification

**Figure 3.**
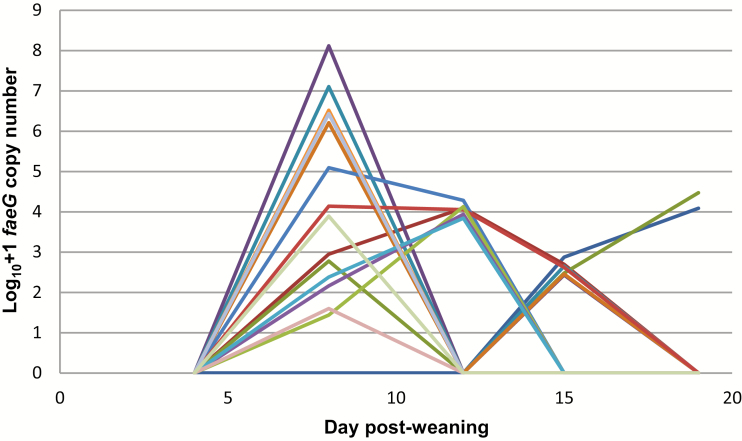
ETEC quantification in ETEC-exposed pigs by qPCR: Fecal shedding of ETEC over the trial duration for each ETEC-exposed pig pre-exposure (day 4) and postexposure (*n* = 15, 1 pig missed out of analysis due to missing value). ETEC = enterotoxigenic *Escherichia coli*; qPCR = quantitative PCR.

All fecal samples from control pigs were confirmed as ETEC F4 negative by qPCR. The dynamics of ETEC excretion were expressed over the trial period as log transformed (Log_10_ + 1) *faeG* gene copy number per gram of wet feces (Fig. 3). On day 4 (pre-exposure), all pigs tested negative when targeting the *faeG* gene. On day 8 postweaning, the highest levels of ETEC excretion were observed with 14 of 15 exposed pigs shedding detectable levels of ETEC F4. Excretion levels dropped over the remaining sampling points with the number of pigs having detectable levels of ETEC in their feces reducing over time.

### ETEC Exposure and Fecal Microbiota Composition

Although there was an indication that a decrease in richness occurred more rapidly in ETEC-exposed pigs in comparison with the control pigs (RM-ANOVA: *P* = 0.05), the highly significant decrease in richness and diversity over time was not affected overall by ETEC exposure (RM-ANOVA: *P* > 0.05; Table 2).

The statistical significance of clustering in relation to ETEC excretion status was assessed. First, it was confirmed that there were no significant differences in community structure when comparing ETEC-exposed and control pigs at baseline (AMOVA: *P* = 0.65). Second, there were no significant differences in community structure at any of the sampling points postexposure when comparing pigs from both treatment groups (AMOVA: *P* > 0.05).

The stabilities of ETEC-exposed and control pig fecal communities were also compared at each sampling point, and no significant effects of ETEC exposure were observed (HOMOVA: *P* > 0.05). In addition, there were no differentially expressed OTUs over the duration of the experiment when comparing ETEC- and sham-exposed pigs (ANCOM: *P* > 0.05).

### ETEC Shedding Variation and Microbiota Composition

Large variation was observed in ETEC F4 excretion level across all exposed pigs. To explore potential links between microbiota composition and ETEC shedding levels, all pigs exposed to ETEC were split into various groups based on the qPCR data, and 2 separate analyses were run.

**Figure 4.**
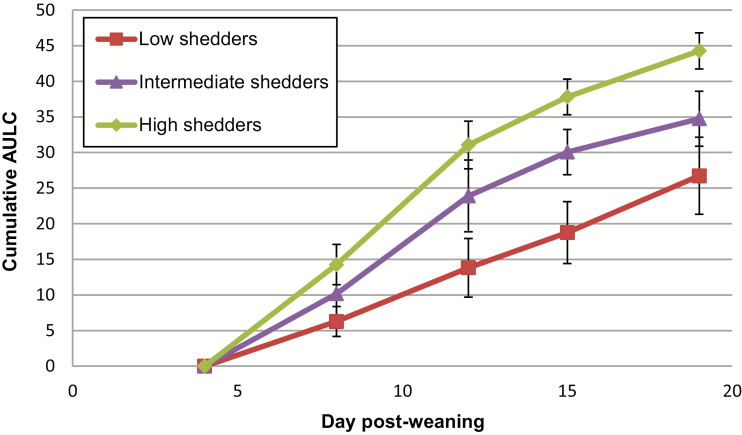
Variation in ETEC shedding levels: Cumulative AULC representing ETEC shedding level (i.e., low, intermediate, and high shedding) by ETEC-exposed pigs (± SD). The cumulative AULC was calculated using log normalized data for each pig at each of the sampling points postweaning. AULC = area under the log curve; ETEC = enterotoxigenic *Escherichia coli*.

First, the cumulative area under the log curve (**AULC**) was calculated (Fig. 4). Five “low shedders” and 5 “high shedders” were selected, based on their presence within the lower and upper ranges of the AULC data. At baseline (day 4) and at all time points postexposure, there were no differences in community structure (AMOVA: *P* > 0.05), stability (HOMOVA: *P* > 0.05), or phylotype relative abundances (Metastats: *P* > 0.05) when considering ETEC excretion level. However, on day 8, the high shedders had a significantly different community structure in comparison to the control pigs (AMOVA: *P* = 0.013), whereas the low shedders had a more similar community structure to the control pigs (AMOVA: *P* > 0.05), with no associated changes in phylotype relative abundances (Metastats: *P* > 0.05). This community structure difference was not present for the remainder of the experiment (AMOVA: *P* > 0.05). In addition, there were no consistently differentially expressed phylotypes when comparing low and high shedders (ANCOM: *P* > 0.05).

**Figure 5.**
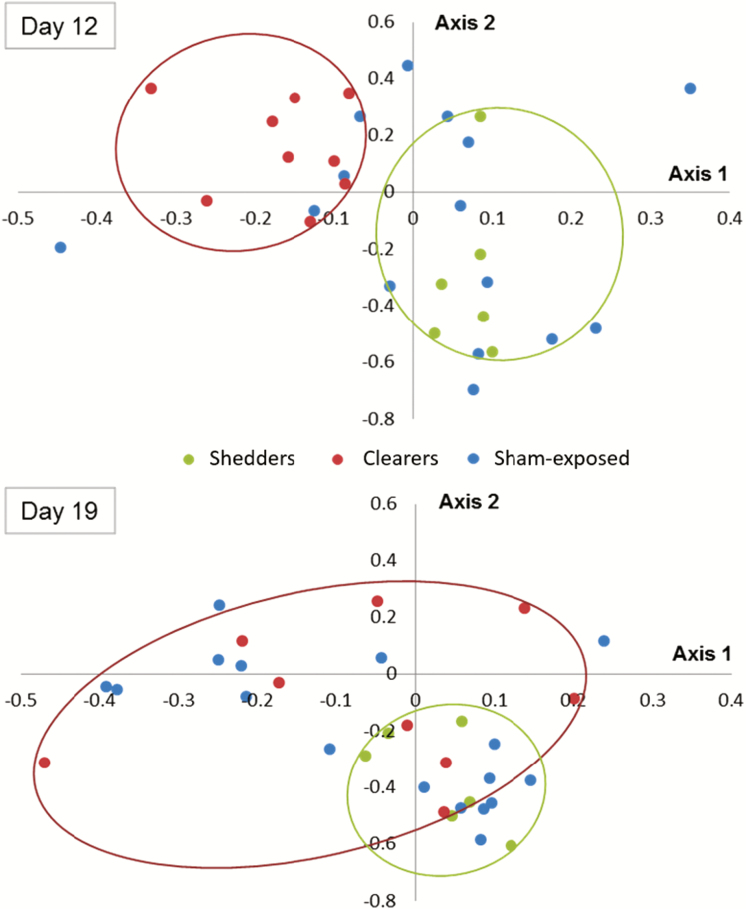
NMDS ordination of comparing fecal communities on days 12 and 19 by shedding status: Community structures were significantly different on days 12 (AMOVA: *P* = 0.029) and 19 (AMOVA: *P* = 0.037) based on shedding dynamics in day 12, where 2 distinct ETEC “clearer” and “shedder” groups were observed. On day 19, the pigs shedding no detectable levels of ETEC had more variable bacterial communities in comparison with the pigs shedding ETEC in the feces (HOMOVA: *P* = 0.045). AMOVA = analysis of molecular variance; ETEC = enterotoxigenic *Escherichia coli*; HOMOVA = homogeneity of molecular variance; NMDS = non-metric multidimensional scaling.

Second, to investigate a clear contrast in shedding dynamics on day 12 (Fig. 3), pigs were then retrospectively clustered into 2 groups—ETEC “clearers” (i.e., no shedding detected, *n* = 9) and ETEC “shedders” (i.e., shedding evident between 6.95 × 10^2^ and 1.91 × 10^3^ *faeG* copies/g wet feces, *n* = 6). No differences in community structure were evident in these groups prior to ETEC exposure on day 4 (AMOVA: *P* > 0.05), but the community structures were significantly different on days 12 (AMOVA: *P* = 0.029) and 19 (AMOVA: *P* = 0.037), as shown in Fig. 5, but were not significantly different on days 8 and 15 (AMOVA: *P* > 0.05). Although there were observed changes in community structure, these were not linked with significant changes in relative abundance of particular bacterial phylotypes (ANCOM: *P* > 0.05, Metastats: *P* > 0.05). There were also significant differences in community stabilities on day 19, whereby the ETEC clearers had more variable bacterial communities in comparison with the ETEC shedders (HOMOVA: *P* = 0.045). No differences in community stability were observed on days 4, 8, 12, and 15 (HOMOVA: *P* > 0.05).

### Growth Rate and Fecal Consistency Scores

The mean BW for pigs included in the study and the subset selected for 16S rRNA gene metabarcoding are presented in Table 4. There were no significant effects of ETEC exposure on either BW (RM-ANOVA: *P* = 0.63) or ADG (ANOVA: *P* = 0.13) when considering the total population of pigs (ETEC exposed, *n* = 32; control, *n* = 27). When considering the pigs that were subject to 16S rRNA gene metabarcoding only (ETEC exposed, *n* = 16; control, *n* = 16), there were again no significant effects of ETEC exposure on BW (RM-ANOVA: *P* = 0.80) or ADG (ANOVA: *P* = 0.56).

**Table 4.** Mean BW for all ETEC-exposed (ETEC) and sham-exposed (Sham) pigs included in this study (all) and for pigs selected for 16S rRNA gene metabarcoding only (16S)

	Mean BW, kg ± SD				
Group	Day 0	Day 7	Day 14	Day 21	Day 28
ETEC (all)	8.59 ± 1.52	10.01 ± 1.63	13.23 ± 1.90	17.94 ± 2.39	23.43 ± 3.00
ETEC (16S)	8.71 ± 1.39	10.29 ± 1.62	13.33 ± 1.93	18.01 ± 2.57	23.43 ± 3.26
Sham (all)	8.82 ± 1.77	10.12 ± 2.15	13.24 ± 2.67	18.33 ± 3.47	24.34 ± 4.14
Sham (16S)	8.80 ± 1.99	10.14 ± 2.43	13.32 ± 3.21	18.15 ± 4.23	24.20 ± 5.12

**Figure 6.**
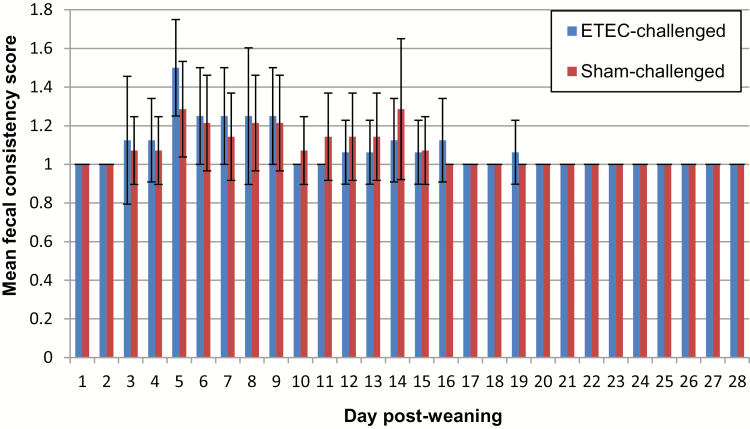
Mean fecal consistency scores: Bar graph showing the mean fecal consistency scores (± SD) in both ETEC- and sham-exposed pens from days 1 to 28 postweaning, with no significant effects of subclinical ETEC exposure being observed (*P* > 0.05). ETEC = enterotoxigenic *Escherichia coli*.

Mean fecal consistency scores for both ETEC-exposed and control pens throughout the trial are presented in Fig. 6. Overall, there was not a statistically significant effect of ETEC exposure on fecal score (OLR: *P* = 0.822), but statistically significant temporal effects on fecal consistency score were found (OLR: *P* < 0.001). On days 3 and 4 (pre-exposure), slightly elevated mean fecal consistency scores were observed in both ETEC-exposed and control pens. On day 5 (i.e., 1-d postexposure), the mean fecal score in the ETEC-exposed pens increased to 1.5 ± 0.25 and 1.29 ± 0.25 in the control pens. After day 5, the fecal scores gradually decreased, and by day 20, all pens were assigned a fecal consistency score of 1. For the remaining 8 d of the trial, all pens were consistently assigned a fecal score of 1.

## DISCUSSION

The primary aim of this work was to study potential effects of subclinical ETEC exposure in pigs on fecal microbiota composition, with a secondary aim of studying temporal shifts in bacterial communities over the weaning transition period.

### Sequencing Controls

Contamination can arise from personnel and laboratory consumables, as well as DNA extraction kits and PCR reagents, which can present during data analysis (Salter et al., 2014). In addition, it is good practice to include a known mock bacterial community to assess the methodology used for microbiome sequencing experiments. As a consequence, the inclusion of sequencing controls has been strongly encouraged (Pollock et al., 2018). In this study, low DNA yield was observed in the reagent-only control extracts and background DNA contamination was evident postanalysis. The sequences generated were diverse and low read numbers were observed within each phylotype. Because fecal samples have a high biomass and are less sensitive to contamination biases, phylotypes that were identified in fecal samples and the reagent-only controls were not removed from the analysis.

When comparing the expected relative abundances with the measured relative abundances in the mock community, it was found that some bacterial species were over-represented or under-represented by this method. This misrepresentation may be caused by a variety of factors, including primer biases and the bioinformatics pipeline used (Schloss et al., 2011; Pinto and Raskin, 2012). Indeed, the calculated error rate as part of this sequencing run was low (i.e., 0.03%), which ensured that overinflation of bacterial diversity due to sequencing error would have been minimized.

### Temporal Changes in the Fecal Microbiota

Over 80% of all DNA sequences generated in this study belonged to the Bacteroidetes and Firmicutes phyla, which is in line with existing literature (Kim et al., 2011; Costa et al., 2014; Mach et al., 2015; Hu et al., 2016; Chen et al., 2017). An increase in Bacteroidetes was evident over the immediate postweaning period, which is also reflected in other work (Pajarillo et al., 2014; Hu et al., 2016; Chen et al., 2017). This increase in Bacteroidetes was clearly driven by increases in Prevotellaceae, which contains the *Prevotella* genus. Similarly, as in other previous work, *Prevotella* was the most dominant genus found in the fecal samples (Kim et al., 2011, 2012; Lamendella et al., 2011; Liu et al., 2012; Looft et al., 2012; Holman and Chénier, 2014; Hu et al., 2016; Chen et al., 2017) and increases in relative abundance as the pig ages (Pajarillo et al., 2014; Mach et al., 2015; Hu et al., 2016; Chen et al., 2017). The increase in *Prevotella* after weaning is probably due to the ability of this bacterium to produce enzymes that can break down polysaccharides in the cereal cell wall (Flint et al., 2008).

Significant changes in microbiota structure (i.e., bacterial membership and associated relative abundances) were observed over the immediate postweaning period. Previous work has established that suckling piglets have different gut microbial communities in comparison with weaner piglets (Kim et al., 2011; Holman and Chénier, 2014; Pajarillo et al., 2014; Zhao et al., 2015). These observed community shifts postweaning are most likely associated with the piglets’ removal from a highly digestible milk source, the consequent removal of maternal antibodies, and sudden introduction to a less digestible, solid, plant-based diet (Lallès et al., 2007). The weaning process is also linked with social stressors, such as separation from the sow and litter mixing, which contributes to greater susceptibility to enteric disorders (Lallès et al., 2007). In addition, the environment in which a piglet is housed has an impact on mucosal immune function and microbiota composition (Mulder et al., 2009) and will probably have a collective impact on the gut microbiota structure.

In this study, the fecal microbiota became more stable over the weaning transition period. Differences in community stability have been linked with healthy and unhealthy states, and temporal microbiota studies assist in linking variations in stability with variation in health states (Schloss et al., 2012). Throughout the duration of this experiment, all pigs remained clinically healthy, which may assist in explaining why the fecal microbiota moved toward a more stable composition in a relatively short time-frame postweaning.

### ETEC Excretion Level and the Fecal Microbiota

Although there were no main effects of ETEC exposure on temporal fecal microbiota structure, stability, or relative phylotype levels, there were considerable variations in ETEC excretion levels indicative of variable gastrointestinal tract colonization (Hampson et al., 1985; Geenen et al., 2007). Specifically, on day 12, there were 2 clear groupings of pigs defined as “ETEC clearers” and “ETEC shedders.” The latter group showed a drastic increased in ETEC shedding from the previous time point, which may be explained by better adhesion and/or colonization of ETEC F4 in the small intestine (Geenen et al., 2007). Pigs that were more resistant to ETEC exposure may have shown lower excretion levels of this bacterium, which would represent the time the organism took to pass through the pig without significant interactions with the gastrointestinal tract (Hil et al., 2017). This level of variation between individuals has been shown in previous studies on ETEC exposure (Hil et al., 2017) and *Salmonella* exposure (Bearson et al., 2013) and may be attributable to the health status of the animals and genetic variability underpinning innate and adaptive immune responses. It is also proposed that competition with the commensal microbiota may play an important role in ETEC colonization and excretion dynamics.

In this study, ETEC excretion level was associated with changes in fecal microbiota structure and stability at specific sampling points. On day 8, when taking cumulative ETEC shedding levels into account, the high shedders had a significantly different community structure in comparison with the control animals. In addition, the low shedders had a more similar community structure to the control animals rather than the high shedders at this time point. Although structural differences were observed, there were no temporal differences in phylotype relative abundances when comparing low and high shedders, suggesting that no specific phylotypes were correlated with ETEC shedding variation. As previously described, there were 2 clear groupings evident in terms of shedding levels on day 12, and when animals were retrospectively clustered into these groups, their community structures were significantly different on days 12 and 19. Although structural differences were observed on days 12 and 19, these were not significantly linked with specific phylotypes, potentially suggesting that several relative abundance shifts are occurring across many taxa. Furthermore, higher fecal microbial diversity was observed in pigs that had no measurable ETEC in the feces (“clearers”) in comparison to pigs excreting ETEC (“shedders”) on day 19. Similarity to the low and high shedder pigs, there were no specific phylotypes that correlated with this large variation in ETEC shedding level. Collectively, we consider that the study does provide evidence of a relationship between fecal microbiota composition and ETEC excretion level.

In conclusion, a profound change in the fecal microbiota of pigs was observed over the short weaning transition period, with evidence of a relationship between ETEC excretion and fecal microbiota structure and stability being revealed.

## SUPPLEMENTARY DATA

Supplementary data are available at *Journal of Animal Science* online.

Supplementary Appendices
