# Supplementary material for: Analysis of temporal fecal microbiota dynamics in weaner pigs with and without exposure to enterotoxigenic Escherichia coli
Source: J Anim Sci. 2018 Jul 3;96(9):3777–90. doi: 10.1093/jas/sky260 (PMC6127793; doi:10.1093/jas/sky260)
Supplement: Supplementary Appendices [file sky260_suppl_supplementary_appendices.docx]

**APPENDICES**

**Appendix 1. Information that was considered upon selection of particular pigs for recruitment to the 16S rRNA gene metabarcoding study.**

| **Pig ID** | **Sequencing ID** | **Litter ID** | **Sex** | **Treatment** | **Pen ID** | **Round** | **Weaning weight (kg)** |
| --- | --- | --- | --- | --- | --- | --- | --- |
| 2780 | 1 | BF208 | Female | Infect | Pen 1 | Round 1 | 7.48 |
| 2756 | 2 | BF219 | Male | Infect | Pen 2 | Round 1 | 10.24 |
| 2768 | 3 | BF219 | Female | Infect | Pen 3 | Round 1 | 8.90 |
| 2782 | 4 | BF208 | Male | Infect | Pen 4 | Round 1 | 8.36 |
| 843 | 5 | 36 | Female | Infect | Pen 5 | Round 2 | 6.40 |
| 795 | 6 | 1693 | Female | Infect | Pen 6 | Round 2 | 6.90 |
| 847 | 7 | 36 | Male | Infect | Pen 7 | Round 2 | 9.20 |
| 763 | 8 | 816 | Male | Infect | Pen 8 | Round 2 | 7.80 |
| 2749 | 9 | BF212 | Female | Infect | Pen 1 | Round 1 | 9.14 |
| 2722 | 10 | BF205 | Male | Infect | Pen 2 | Round 1 | 8.30 |
| 2779 | 11 | BF208 | Male | Infect | Pen 3 | Round 1 | 6.66 |
| 2740 | 12 | BF297 | Male | Infect | Pen 4 | Round 1 | 7.14 |
| 759 | 13 | 137 | Female | Infect | Pen 5 | Round 2 | 8.80 |
| 861 | 14 | 125 | Female | Infect | Pen 6 | Round 2 | 10.30 |
| 860 | 15 | 125 | Female | Infect | Pen 7 | Round 2 | 11.20 |
| 785 | 16 | 803 | Male | Infect | Pen 8 | Round 2 | 10.60 |
| 2777 | 17 | BF208 | Female | Sham | Pen 9 | Round 1 | 4.90 |
| 2774 | 18 | BF210 | Male | Sham | Pen 10 | Round 1 | 4.80 |
| 2750 | 19 | BF212 | Female | Sham | Pen 11 | Round 1 | 11.00 |
| 2718 | 20 | BF205 | Male | Sham | Pen 9 | Round 1 | 9.36 |
| 800 | 21 | 1693 | Male | Sham | Pen 12 | Round 2 | 8.20 |
| 827 | 22 | 454 | Female | Sham | Pen 13 | Round 2 | 6.40 |
| 789 | 23 | 803 | Female | Sham | Pen 14 | Round 2 | 7.50 |
| 768 | 24 | 816 | Male | Sham | Pen 15 | Round 2 | 8.00 |
| 2735 | 25 | BF299 | Male | Sham | Pen 9 | Round 1 | 8.42 |
| 2766 | 26 | BF219 | Female | Sham | Pen 10 | Round 1 | 10.52 |
| 2741 | 27 | BF297 | Male | Sham | Pen 11 | Round 1 | 11.00 |
| 2752 | 28 | BF212 | Female | Sham | Pen 9 | Round 1 | 10.22 |
| 820 | 29 | 126 | Female | Sham | Pen 12 | Round 2 | 9.90 |
| 837 | 30 | 459 | Male | Sham | Pen 13 | Round 2 | 10.10 |
| 838 | 31 | 459 | Male | Sham | Pen 14 | Round 2 | 9.70 |
| 863 | 32 | 125 | Male | Sham | Pen 15 | Round 2 | 11.70 |

**Appendix 2.** Primers including Illumina adapters and unique barcodes for sequencing.

| Primer | Sequence (5’-3’) |
| --- | --- |
| 341-F1 | AATGATACGGCGACCACCGAGATCTACACTATAGCCTACACTCTTTCCCTACACGACGCTCTTCCGATCTNNNNCCTACGGGAGGCAGCAG |
| 341-F2 | AATGATACGGCGACCACCGAGATCTACACATAGAGGCACACTCTTTCCCTACACGACGCTCTTCCGATCTNNNNCCTACGGGAGGCAGCAG |
| 341-F3 | AATGATACGGCGACCACCGAGATCTACACCCTATCCTACACTCTTTCCCTACACGACGCTCTTCCGATCTNNNNCCTACGGGAGGCAGCAG |
| 341-F4 | AATGATACGGCGACCACCGAGATCTACACGGCTCTGAACACTCTTTCCCTACACGACGCTCTTCCGATCTNNNNCCTACGGGAGGCAGCAG |
| 341-F5 | AATGATACGGCGACCACCGAGATCTACACAGGCGAAGACACTCTTTCCCTACACGACGCTCTTCCGATCTNNNNCCTACGGGAGGCAGCAG |
| 341-F6 | AATGATACGGCGACCACCGAGATCTACACTAATCTTAACACTCTTTCCCTACACGACGCTCTTCCGATCTNNNNCCTACGGGAGGCAGCAG |
| 341-F7 | AATGATACGGCGACCACCGAGATCTACACCAGGACGTACACTCTTTCCCTACACGACGCTCTTCCGATCTNNNNCCTACGGGAGGCAGCAG |
| 341-F8 | AATGATACGGCGACCACCGAGATCTACACGTACTGACACACTCTTTCCCTACACGACGCTCTTCCGATCTNNNNCCTACGGGAGGCAGCAG |
| 518-R1 | CAAGCAGAAGACGGCATACGAGATCGAGTAATGTGACTGGAGTTCAGACGTGTGCTCTTCCGATCTNNNNATTACCGCGGCTGCTGG |
| 518-R2 | CAAGCAGAAGACGGCATACGAGATTCTCCGGAGTGACTGGAGTTCAGACGTGTGCTCTTCCGATCTNNNNATTACCGCGGCTGCTGG |
| 518-R3 | CAAGCAGAAGACGGCATACGAGATAATGAGCGGTGACTGGAGTTCAGACGTGTGCTCTTCCGATCTNNNNATTACCGCGGCTGCTGG |
| 518-R4 | CAAGCAGAAGACGGCATACGAGATGGAATCTCGTGACTGGAGTTCAGACGTGTGCTCTTCCGATCTNNNNATTACCGCGGCTGCTGG |
| 518-R5 | CAAGCAGAAGACGGCATACGAGATTTCTGAATGTGACTGGAGTTCAGACGTGTGCTCTTCCGATCTNNNNATTACCGCGGCTGCTGG |
| 518-R6 | CAAGCAGAAGACGGCATACGAGATACGAATTCGTGACTGGAGTTCAGACGTGTGCTCTTCCGATCTNNNNATTACCGCGGCTGCTGG |
| 518-R7 | CAAGCAGAAGACGGCATACGAGATAGCTTCAGGTGACTGGAGTTCAGACGTGTGCTCTTCCGATCTNNNNATTACCGCGGCTGCTGG |
| 518-R8 | CAAGCAGAAGACGGCATACGAGATGCGCATTAGTGACTGGAGTTCAGACGTGTGCTCTTCCGATCTNNNNATTACCGCGGCTGCTGG |
| 518-R9 | CAAGCAGAAGACGGCATACGAGATCATAGCCGGTGACTGGAGTTCAGACGTGTGCTCTTCCGATCTNNNNATTACCGCGGCTGCTGG |
| 518-R10 | CAAGCAGAAGACGGCATACGAGATTTCGCGGAGTGACTGGAGTTCAGACGTGTGCTCTTCCGATCTNNNNATTACCGCGGCTGCTGG |
